# Pattern of MRI brain in neuro-psychiatric SLE. Effect of anti-phospholipid antibodies: A study at a tertiary care teaching hospital

**DOI:** 10.12669/pjms.315.7975

**Published:** 2015

**Authors:** Khalid Parvez, Abdul Rahman Saud Al-Arfaj, Muhammad Afzal Hamdani, Faisal Naseeb, Abdulkader Daif, Sajjad Hussain

**Affiliations:** 1 Khalid Parvez, FCPS, MRCP. Registrar, Division of Rheumatology, Department of Medicine, King Khalid University Hospital (KKUH), King Saud University (KSU), Riyadh, Saudi Arabia; 2 Abdul Rahman Saud Al-Arfaj, MRCP, FRCPC, ABIM, Professor of Rheumatology, Department of Medicine, King Khalid University Hospital (KKUH), King Saud University (KSU), Riyadh, Saudi Arabia; 3 Muhammad Afzal Hamdani, FCPS, Dip-Card., MRCP. Senior Registrar, Division of Rheumatology, Department of Medicine, King Khalid University Hospital (KKUH), King Saud University (KSU), Riyadh, Saudi Arabia; 4 Faisal Naseeb, FCPS, MRCP. Registrar, Division of Rheumatology, Department of Medicine, King Khalid University Hospital (KKUH), King Saud University (KSU), Riyadh, Saudi Arabia; 5 Dr. Abdulkader Daif, MD. Professor of Neurology, Department of Medicine, King Khalid University Hospital (KKUH), King Saud University (KSU), Riyadh, Saudi Arabia; 6 Sajjad Hussain MD. Assistant Professor and Consultant Neuro-radiologist, Department of Radiology, King Khalid University Hospital (KKUH), King Saud University (KSU), Riyadh, Saudi Arabia

**Keywords:** Systemic lupus erythematosus, Antiphospholipid antibodies, White matter hyperintensities, Magnetic resonance imaging, Lupus anticoagulant, venous sinus thrombosis

## Abstract

**Objective:**

To compare the neuro-radiologic findings in Systemic lupus erythematosus (SLE) patients with and without antiphospholipid antibodies (aPL) in different neuro-psychiatric manifestations.

**Methods:**

This cross-sectional comparative study was carried out at King Khalid University Hospital, a tertiary care teaching hospital, Riyadh, Saudi Arabia from June 2012 to January 2015. Ninety seven SLE patients with neuro-psychiatric manifestations were included in the study and divided into two groups. Group I (50 patients) SLE with aPL and group II (47 patients) SLE without aPL. We compared Demographic features, clinical manifestations and magnetic resonance imaging (MRI) brain findings.

**Results:**

Demographic and clinical characteristics of two groups were similar. In Group-I, anticardiolipin antibodies (aCL) were most common (86%). In patients with headache, most of the patients in Group-I had white matter hyperintensities (WMHIs) (50% vs 27%) while most of the patients in Group-II had normal MRI brain (38% vs 73%). Similarly WMHIs were found more in Group-I patients with seizures (60% vs 21%), while ischemia/infarction, atrophy and normal MRI were found in Group-II. MRI brain in patients with neurological deficit and psychiatric disorder were not much different in both the groups.

**Conclusion:**

We found no statistically significant differences in frequencies of MRI brain abnormalities in SLE patients with and without aPL antibodies. Each of the three aPL may have a variable effect on the brain.

## INTRODUCTION

Systemic lupus erythematosus (SLE) is a multi-organ autoimmune disease that primarily affects women of childbearing-age. Neurological involvement in SLE is mainly manifested as cerebrovascular accidents, seizures, cognitive impairment, headache and psychosis.^1^ In Saudi Arabia prevalence of SLE is estimated to be 19.28 per 100,000 population.^2^ In a retrospective analysis over a 10-year period, 51% of SLE patients were found to have neuropsychiatric manifestations.^3^ A study conducted by Al-Arfaj et al. showed neuropsychiatric manifestations in 27.6% of SLE patients in Saudi Arabia.^4^ Antiphospholipid antibodies (aPL) are a heterogeneous family of antibodies to phospholipids and phospholipid-binding proteins. The most clearly established clinical manifestations associated with these antibodies are related to thrombosis and recurrent fetal loss. Venous thrombosis is the most common thrombotic manifestation established with aPL; however, cerebrovascular thrombosis is the most common arterial thrombotic manifestation.^5^

anticardiolipin antibodies (aPL) have been associated with the impairment of the normal inhibition of cerebral at herogenesis, leading to an increase in atherosclerotic vascular disease in the brain. aPL mediated complement activation in the brain is also a potential source of nervous system toxicity and dysfunction.^6^ These antibodies were present in 61% of SLE patients and in about half of whom the presence of aPL was associated with clinical thrombotic complication. Among those patients who did not have aPL (*n*-107), 24 had a thrombotic event. This difference in prevalence of thrombosis between patients with and without aPL antibody was significant (51% versus 22.4%; *P* <0.002).^7^

MRI is very important tool for the non-invasive assessment of neurological manifestations of SLE. No sufficient data is available to compare MRI brain findings in different neuro-psychiatric manifestations in SLE patients with and without aPL. The objective of our study was to uncover the pattern of abnormalities seen on conventional MRI in a series of SLE patients (with & without aPL antibodies) presenting with neuro-psychiatric manifestations.

## METHODS

This cross-sectional comparative study was carried out at King Khalid University Hospital Riyadh, Saudi Arabia from June 2012 to January 2015. The study was approved by the ethical committee of our institution. Ninety seven SLE patients attending rheumatology out-patient clinic or admitted with neuro-psychiatric manifestations, were included in the study. All of them fulfilled the American College of Rheumatology criteria. They were divided into two groups, Group-I, SLE with aPL antibodies (n=50) and Group-II, SLE without aPL antibodies (n=47) patients. Informed consent was obtained for inclusion in the study. Data was collected by filling the questionnaire. History, physical examination and lab work including serological profiling were recorded. aCL antibodies and where necessary lupus anticoagulant (LA) andanti-ß2GP1 were obtained. Patients underwent brain MRI on different MR machines including 3.0 Tesla Siemens Vario, 1.5 Tesla GE Discovery 450 and 1.5 Tesla GE Optima 450W. T1-weighted, T2-weighted, fluid-attenuated inversion recovery (FLAIR) and diffusion weighted images were obtained for all the patients. MRI examinations were studied by neuro-radiologist and their clinical data were correlated with the radiological findings by a team comprising of rheumatologist, neurologist and neuro-radiologist. Descriptive statistics (means, standard deviation, and percentages) were used to describe the quantitative and categorical study variables. Chi- square statistics and the Fisher’s exact test were used for categorical data. A two-sided p <0.05 was considered statistically significant. SPSS version 18 (SPSS inc. Chicago, IL, USA) was used for all analysis.

## RESULTS

Ninety seven SLE patients were recruited with a mean age of 37.55 ± 11.218 years (range 15-68). Ninety (90/97) patients were female and seven (7/97) were male. Fifty SLE patients in Group-I were positive and forty seven (Group-II) were negative for aPL. There was no significant difference between the two groups regarding demographic features. In group I, aCL antibodies were most common, which were found in 43(86%), followed by lupus anticoagulant in 16(32%) and anti-ß2GP1 in 5(10%) of SLE patients. Eleven patients were positive for both aCL and LA, 1 had aCL and anti-ß2GP1 and 2 patients had both LA and anti-ß2GP1 in their serum.

The common neuro-psychiatric manifestations in our study were headache, seizures, neurological deficit and psychiatric disorders (depression, psychosis, cognitive impairment or mood disorder). Headache was found in 16(32%) patients of group one followed by seizures in 10(20%), neurological deficit in 14(28%) and psychiatric disorder in 10(20%) patients. In group II, 11(23%) patients had headache, 14(30%) seizures, 12(26%) neurological deficit and 10(21%) had some psychiatric disorder.

Differences in the MRI abnormalities in the two groups are presented in Table-I. Common findings in Group-I were WMHIs 20(40%), infarction 12(24%), venous sinus thrombosis (VST) and atrophy 1(2%) and 16(32%) patients had normal MRI. WMHIs in Group-II were found in 11(23%) patients followed byischemia/infarction in 10(21%), brain atrophy 6(13%) and no abnormality was found in 20(42%) patients. No patient had VST in Group-II.

**Table-I:** Comparison between SLE patients with or without aPL.

	SLE patients with aPL (n=50)	SLE patients without aPL (n=47)	p-value
Age	38.5±9.9	36.5±12.4	0.381
Sex, M/F	5/45	2/45	0.437
Presentation			
Headache	16 (32)	11 (23)	0.345
Seizures	10 (20)	14 (30)	0.264
Neurologic deficit	14 (28)	12 (26)	0.784
Psychiatric disorder	10 (20)	10 (21)	0.877
MRI findings			
White matter	20 (40)	11 (23)	0.080
Ischemia/infarction	12 (24)	10 (21)	0.749
Venous thrombosis	1 (2)	0	1.000
Brain atrophy	1 (2)	6 (13)	0.054
Normal	16 (32)	20 (42)	0.282
Serology			
Anticardiolipin antibodies	43 (86)	0	<0.0001
Lupus anticoagulant	16 (32)	0	<0.0001
β2-glycoprotein	5 (10)	0	0.069

**Table-II:** Headache and MRI findings among SLE patients with and without aPL.

	SLE patients with APS (n=16)	SLE patients without APS (n=11)	p-value
MRI findings			
White matter, changes	8 (50)	3 (27)	0.238
Ischemia/infarction, changes	2 (12)	0	0.499
Venous thrombosis	-	-	-
Brain atrophy	-	-	-
Normal	6 (38)	8 (73)	0.072
Seizures and MRI findings among SLE patients with and without aPL.			

	SLE patients with APS (n=16)	SLE patients without APS (n=11)	p-value
MRI findings			
White matter, changes	6 (60)	3 (21)	0.054
Ischemia/infarction, changes	3 (30)	6 (43)	0.521
Venous thrombosis	-	-	-
Brain atrophy	0	2 (14)	0.493
Normal	1 (10)	3 (21)	0.615
Neurologic deficit and MRI findings among SLE patients with and without aPL.			

	SLE patients with APS (n=16)	SLE patients without APS (n=11)	p-value
MRI findings			
White matter, changes	4 (29)	4 (33)	1.000
Ischemia/infarction, changes	6 (43)	4 (33)	0.619
Venous thrombosis	1 (7)	0	1.000
Brain atrophy	0	1 (8)	0.462
Normal	3 (21)	3 (25)	1.000
Psychiatric disorders and MRI findings among SLE patients with and without aPL.			

	SLE patients with APS (n=16)	SLE patients without APS (n=11)	p-value
MRI findings			
White matter, changes	2 (20)	1 (10)	1.000
Ischemia/infarction, changes	1 (10)	0	1.000
Venous thrombosis	-	-	-
Brain atrophy	1 (10)	3 (30)	0.582
Normal	6 (60)	6 (60)	1.000

In Group-I, out of 16 patients with headache, WMHIs were found in 8(50%) patients followed by normal MRI in 6(37.5%) and only 2(12.5%) patients had ischemia/infarcts. No patient had VST or brain atrophy. Among 10 patients presenting with seizures, 6(60%) had WMHIs, 3(30%) ischemia/infarcts and only one had normal MRI brain. Fourteen patients who presented with neurological deficit had following MRI findings: 4(28.6%) had WMHIs, 6(42.9%) infarction and 3(21.4%) had normal MRI. No MRI abnormalities were found in 6(60%) of 10 patients who had psychiatric disorder. Two patients had WMHIs, one had infarction and one had brain atrophy.

Similarly in Group-II, out of 11 patients with headache, WMHIs were found in 3(27.3%) patients while 8(72.7%) had normal MRI. No patient had ischemia/infarction, VST or brain atrophy. Among 14 patients with seizures, 3(21.4%) had WMHIs, 6(42.9%) ischemia/infarcts, 2 brain atrophy and three had normal MRI brain. Twelve patients with neurological deficit had following MRI findings: 4(33.3%) had WMHIs and ischemia/infarction each, 1(8.3%) brain atrophy and 3(25%) had normal MRI. No MRI abnormalities were found in 6(60%) of 10 patients who had psychiatric disorder. Three patients had brain atrophy and one had WMHIs.

## DISCUSSION

Demographic features of our study population are consistent with other areas of the world. No differences were noted between the two groups regarding age and sex of patients. Slightly more than half (51.5%) of our SLE patients with neuropsychiatric manifestations were positive for aPL. In a study done in Bahrain, aCL were detected in 23% while anti-ß2GP1were detected in 16.6% of SLE patients.^8^ In another study, 55% of SLE patients (CNS Lupus) were positive for aPL antibodies.^9^ Sanna G et al. found 60% of their SLE/CNS patients to have aPL. aCL was positive in 76%, LA in 52% and 28% patients of aPL group had both aCL and LA.^10^ So regarding aPL in NPSLE, our findings are in line with other studies.

**Fig.1:**
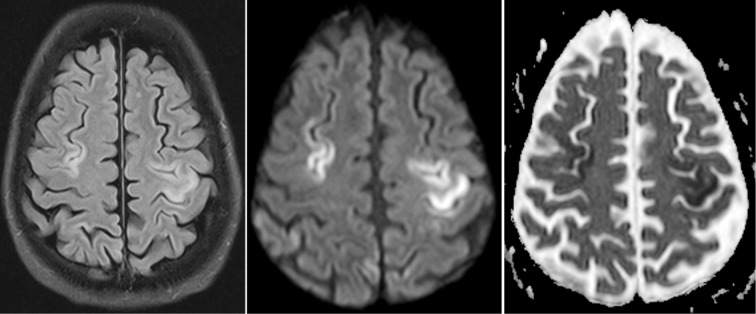
30 year SLE pt. (aPL) with left hemiparesis. Axial FLAIR (left), diffision weighted (middle) and ADC (right) images show multiple foci of diffusion restriction representing acute infarcts in bilateral parietal lobes.

**Fig.2:**
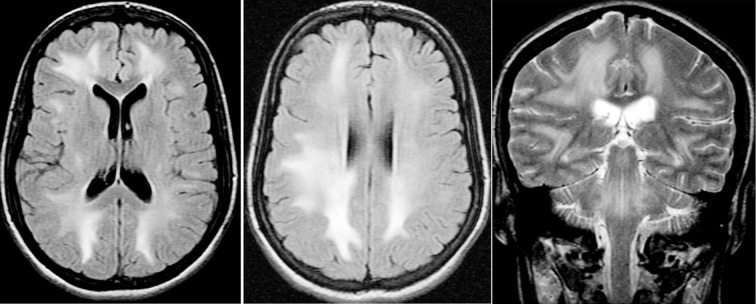
20 year SLE pt. with seizures. Axial FLAIR (A and B) and coronal T2 weighted images show extensive bilateral areas of increased signal intensity in cerebral white matter and brain stem.

Among neuro-psychiatric manifestations; headache, seizures, neurological deficit and psychiatric disorders were the most common symptoms in both the groups. Headache was more common (32% vs 23%; p=0.345) in Group-I as opposed seizures which were more common (20% vs 30%, p=0.264) in Group-II. The frequency of neurological deficits (28% vs 26%) and psychiatric disorders (20% vs 21%) were not much different in the two groups. Sanna G et al. and Alfeltra A et al. reported that headache, CVD, seizures and psychiatric disorders were more common in aPL positive group.^10^,^11^ Similar results were also reported by Brey RL et al except headache which showed no association with aPL.^5^ Sachse C in their study did not find any correlation of aPL with epilepsy and psychosis.^12^ Harris EN et al. in their study on 15 SLE patients with cerebral infarction found aCL in their serum.^13^ Liou et al. found that epilepsy as a neuropsychiatric event among lupus patients is associated with a high titer of aCLantibodies.^14^ Similar findings were also reported by Shrivastava et al. and Appenzeller S et al.^15^,^16^ In a recent study by MA Coin et al, 78% of the SLE patients with aPL showed cognitive deficits, as did 48% of the SLE without aPL.^17^ So neuro-psychiatric manifestations had variable aPL profile as compared to what was observed by other studies.

On MRI brain, WMHIs were observed more in Group-I (40% vs 23%, p=0.080) while brain atrophy was more frequent in group II (2% vs 13%, p=0.054). No differences were observed in the categories of ischemia/infarction (24% vs 21%) and venous sinus thrombosis (2% vs 0). Thirty two percent neuro-psychiatric SLE (NPSLE) patients in Group-I and 42% in Group-II had normal MRI brain (p = 0.282). WMHIs were found more in patients with aPL than those without aPL (36% vs 24%), but the difference was not statistically significant (Sanna G et al).^10^ Toubi E et al. reported 33/53 patients to have WMHIs, of whom 79% were positive for aPL.^9^ Provenzale JM et al. reported abnormal MRI brain in 57% of aPL patients; infarcts and WMHIs being most common, but the difference was not statistically significant.^18^ Thirteen patients had white matter focal brain lesions on MRI, 10 of whom had LAC (p = 0.03) (Molad Y).^19^ Hachulla E et al. did not find any difference on MRI in terms of WMHIs, infarction and brain atrophy in patients with SLE and primary APS.^20^ Herranz MT et al. in their study on SLE patients with seizures reported that all patients with abnormal MRI had positive aPL.^21^

A study by Arinuma Y et al. revealed that 47.2% of SLE patients had abnormal MRI with subcortical WMHIs being the most common finding. However these findings were not significantly associated with aPL.^22^ Appenzeller S et al. reported that hyperintense WM lesions in SLE with central nervous system symptoms are associated with aPL antibodies.^23^ Patients with focal CNS lupus had areas of increased signal intensity and atrophic changes in regions corresponding to the major cerebral vessels. These MRI abnormalities did not improve after treatment with high-dose steroids. The sera of patients with focal CNS lupus had elevated levels of aCL and LA.^24^

In patients with headache, most of the patients in Group-I had WMHIs (50% vs 27%, p=0.238) while most of the patients in Group-II had normal MRI brain (38% vs 73%, p=0.072). Other findings were not significant in patients with headache. Similarly WMHIs were found more in Group-I patients with seizures (60% vs 21%, p=0.054), while more patients in Group-II had ischemia/infarction (30% vs 43%, p=0.521), brain atrophy (0% vs 14%, p=0.493) and normal (10% vs 21%, p=0.615) MRI brain. MRI brain abnormalities in patients presenting with neurological deficit were not much different in both the groups. Same is the case for SLE patients having psychiatric disorder as no significant difference was observed on MRI brain. Csepany T et al. reported that infarcts and atrophy were more frequent in aPL group and were more common in patients with neurological deficit.^25^ No sufficient data is available to compare MRI brain findings in different neuro-psychiatric manifestations in SLE patients with and without aPL. A limitation of our study is the variation in MRI scanners and the consequent inconsistency in the applied pulse sequences. Although the different MRI machines have different Tesla values which would be a factor in resolutions of images but it did not affect interpretation of such images.

## CONCLUSION

We found no statistically significant differences in frequencies of MRI brain abnormalities in SLE patients with and without aPL antibodies. Each of the three aPL may have a variable effect on the brain. Further studies on large scale may be needed to uncover the pattern of MRI abnormalities in SLE for different aPL antibodies.
